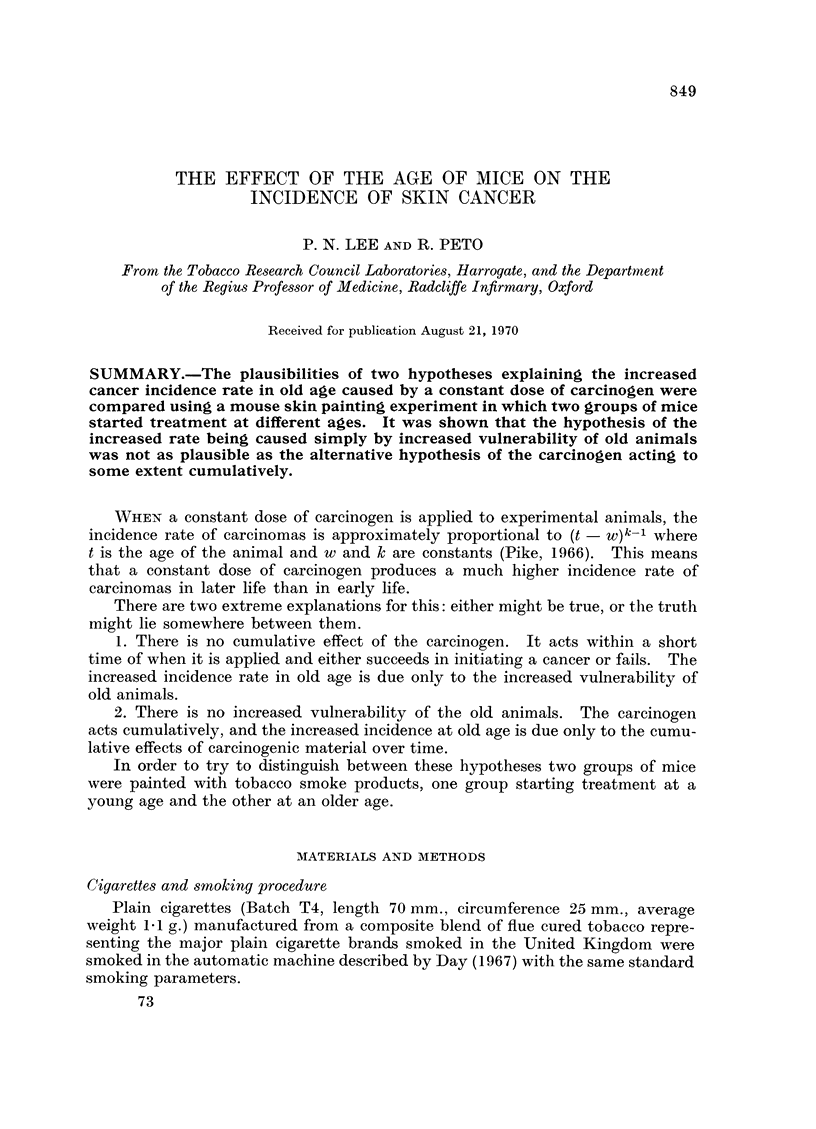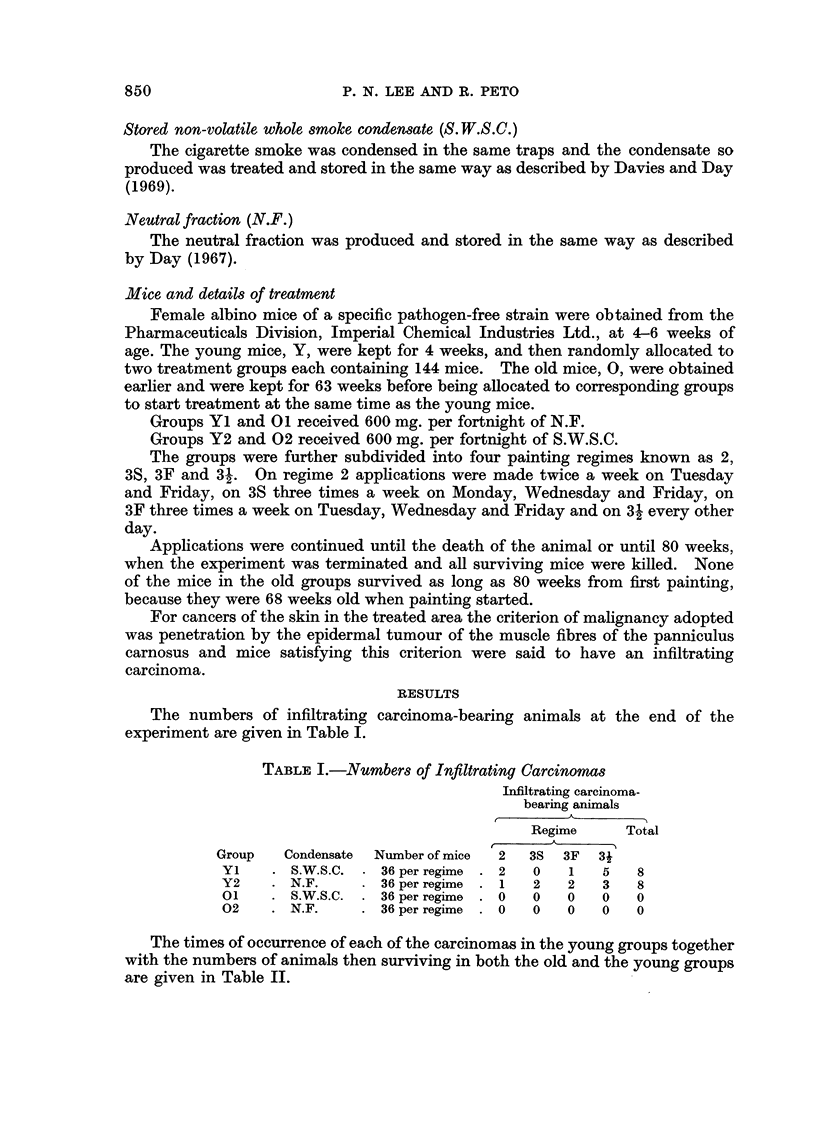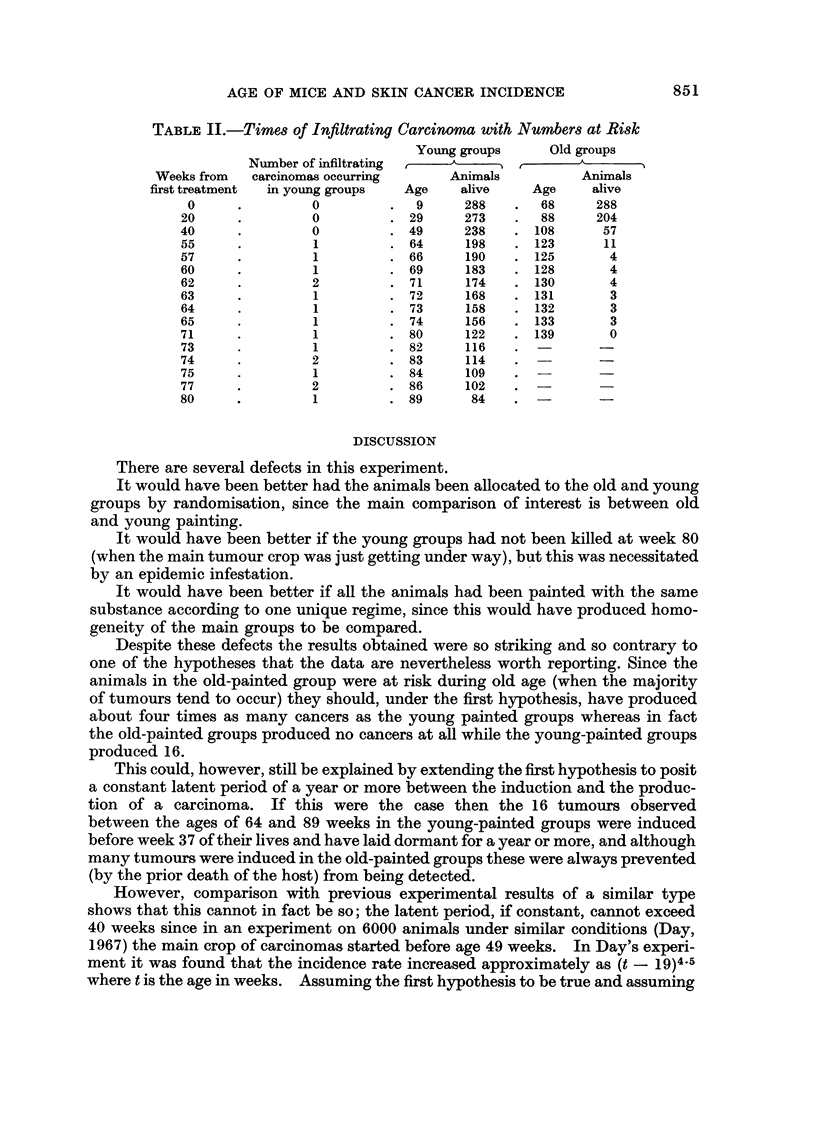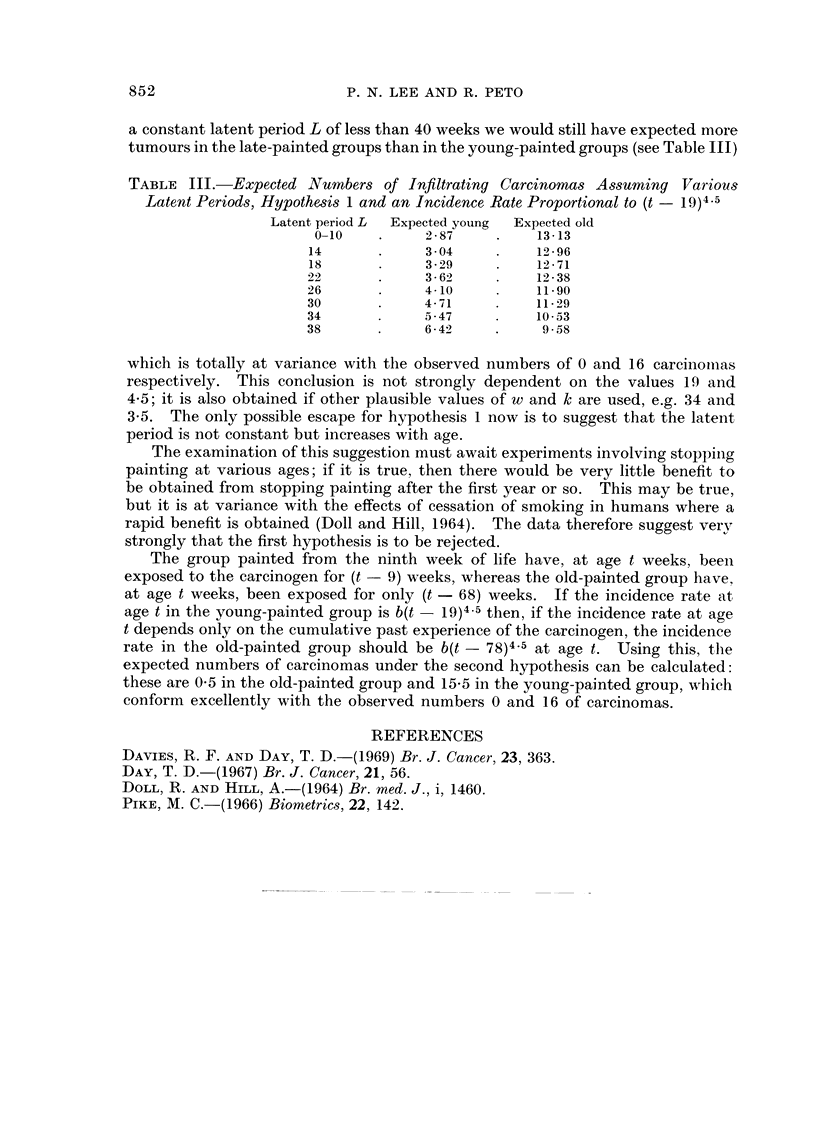# The Effect of the Age of Mice on the Incidence of Skin Cancer

**DOI:** 10.1038/bjc.1970.100

**Published:** 1970-12

**Authors:** P. N. Lee, R. Peto

## Abstract

The plausibilities of two hypotheses explaining the increased cancer incidence rate in old age caused by a constant dose of carcinogen were compared using a mouse skin painting experiment in which two groups of mice started treatment at different ages. It was shown that the hypothesis of the increased rate being caused simply by increased vulnerability of old animals was not as plausible as the alternative hypothesis of the carcinogen acting to some extent cumulatively.


### THE EFFECT OF THE AGE OF MICE ON THE INCIDENCE OF SKIN CANCER

P. N. LEE AND R. PETO

*From the Tobacco Research Council Laboratories, Harrogate, and the Department of the Regius Professor of Medicine, Radcliffe Infirmary, Oxford*

Received for publication August 21, 1970

**SUMMARY.—The plausibilities of two hypotheses explaining the increased cancer incidence rate in old age caused by a constant dose of carcinogen were compared using a mouse skin painting experiment in which two groups of mice started treatment at different ages. It was shown that the hypothesis of the increased rate being caused simply by increased vulnerability of old animals was not as plausible as the alternative hypothesis of the carcinogen acting to some extent cumulatively.**

WHEN a constant dose of carcinogen is applied to experimental animals, the incidence rate of carcinomas is approximately proportional to $(t - w)^{k-1}$ where $t$ is the age of the animal and $w$ and $k$ are constants (Pike, 1966). This means that a constant dose of carcinogen produces a much higher incidence rate of carcinomas in later life than in early life.

There are two extreme explanations for this: either might be true, or the truth might lie somewhere between them.

1. There is no cumulative effect of the carcinogen. It acts within a short time of when it is applied and either succeeds in initiating a cancer or fails. The increased incidence rate in old age is due only to the increased vulnerability of old animals.

2. There is no increased vulnerability of the old animals. The carcinogen acts cumulatively, and the increased incidence at old age is due only to the cumulative effects of carcinogenic material over time.

In order to try to distinguish between these hypotheses two groups of mice were painted with tobacco smoke products, one group starting treatment at a young age and the other at an older age.

### MATERIALS AND METHODS

### *Cigarettes and smoking procedure*

Plain cigarettes (Batch T4, length 70 mm., circumference 25 mm., average weight 1·1 g.) manufactured from a composite blend of flue cured tobacco representing the major plain cigarette brands smoked in the United Kingdom were smoked in the automatic machine described by Day (1967) with the same standard smoking parameters.

*Stored non-volatile whole smoke condensate (S.W.S.C.)*

The cigarette smoke was condensed in the same traps and the condensate so produced was treated and stored in the same way as described by Davies and Day (1969).

*Neutral fraction (N.F.)*

The neutral fraction was produced and stored in the same way as described by Day (1967).

*Mice and details of treatment*

Female albino mice of a specific pathogen-free strain were obtained from the Pharmaceuticals Division, Imperial Chemical Industries Ltd., at 4–6 weeks of age. The young mice, Y, were kept for 4 weeks, and then randomly allocated to two treatment groups each containing 144 mice. The old mice, O, were obtained earlier and were kept for 63 weeks before being allocated to corresponding groups to start treatment at the same time as the young mice.

Groups Y1 and O1 received 600 mg. per fortnight of N.F.

Groups Y2 and O2 received 600 mg. per fortnight of S.W.S.C.

The groups were further subdivided into four painting regimes known as 2, 3S, 3F and 3½. On regime 2 applications were made twice a week on Tuesday and Friday, on 3S three times a week on Monday, Wednesday and Friday, on 3F three times a week on Tuesday, Wednesday and Friday and on 3½ every other day.

Applications were continued until the death of the animal or until 80 weeks, when the experiment was terminated and all surviving mice were killed. None of the mice in the old groups survived as long as 80 weeks from first painting, because they were 68 weeks old when painting started.

For cancers of the skin in the treated area the criterion of malignancy adopted was penetration by the epidermal tumour of the muscle fibres of the panniculus carnosus and mice satisfying this criterion were said to have an infiltrating carcinoma.

### RESULTS

The numbers of infiltrating carcinoma-bearing animals at the end of the experiment are given in Table I.

TABLE I.—*Numbers of Infiltrating Carcinomas*

| | | | Infiltrating carcinoma-bearing animals | | | | |
|---|---|---|---|---|---|---|---|
| | | | Regime | | | | Total |
| Group | Condensate | Number of mice | 2 | 3S | 3F | 3½ | |
| Y1 | S.W.S.C. | 36 per regime | 2 | 0 | 1 | 5 | 8 |
| Y2 | N.F. | 36 per regime | 1 | 2 | 2 | 3 | 8 |
| O1 | S.W.S.C. | 36 per regime | 0 | 0 | 0 | 0 | 0 |
| O2 | N.F. | 36 per regime | 0 | 0 | 0 | 0 | 0 |

The times of occurrence of each of the carcinomas in the young groups together with the numbers of animals then surviving in both the old and the young groups are given in Table II.

TABLE II.—*Times of Infiltrating Carcinoma with Numbers at Risk*

| Weeks from first treatment | Number of infiltrating carcinomas occurring in young groups | Young groups Age | Young groups Animals alive | Old groups Age | Old groups Animals alive |
|---|---|---|---|---|---|
| 0 | 0 | 9 | 288 | 68 | 288 |
| 20 | 0 | 29 | 273 | 88 | 204 |
| 40 | 0 | 49 | 238 | 108 | 57 |
| 55 | 1 | 64 | 198 | 123 | 11 |
| 57 | 1 | 66 | 190 | 125 | 4 |
| 60 | 1 | 69 | 183 | 128 | 4 |
| 62 | 2 | 71 | 174 | 130 | 4 |
| 63 | 1 | 72 | 168 | 131 | 3 |
| 64 | 1 | 73 | 158 | 132 | 3 |
| 65 | 1 | 74 | 156 | 133 | 3 |
| 71 | 1 | 80 | 122 | 139 | 0 |
| 73 | 1 | 82 | 116 | — | — |
| 74 | 2 | 83 | 114 | — | — |
| 75 | 1 | 84 | 109 | — | — |
| 77 | 2 | 86 | 102 | — | — |
| 80 | 1 | 89 | 84 | — | — |

### DISCUSSION

There are several defects in this experiment.

It would have been better had the animals been allocated to the old and young groups by randomisation, since the main comparison of interest is between old and young painting.

It would have been better if the young groups had not been killed at week 80 (when the main tumour crop was just getting under way), but this was necessitated by an epidemic infestation.

It would have been better if all the animals had been painted with the same substance according to one unique regime, since this would have produced homogeneity of the main groups to be compared.

Despite these defects the results obtained were so striking and so contrary to one of the hypotheses that the data are nevertheless worth reporting. Since the animals in the old-painted group were at risk during old age (when the majority of tumours tend to occur) they should, under the first hypothesis, have produced about four times as many cancers as the young painted groups whereas in fact the old-painted groups produced no cancers at all while the young-painted groups produced 16.

This could, however, still be explained by extending the first hypothesis to posit a constant latent period of a year or more between the induction and the production of a carcinoma. If this were the case then the 16 tumours observed between the ages of 64 and 89 weeks in the young-painted groups were induced before week 37 of their lives and have laid dormant for a year or more, and although many tumours were induced in the old-painted groups these were always prevented (by the prior death of the host) from being detected.

However, comparison with previous experimental results of a similar type shows that this cannot in fact be so; the latent period, if constant, cannot exceed 40 weeks since in an experiment on 6000 animals under similar conditions (Day, 1967) the main crop of carcinomas started before age 49 weeks. In Day's experiment it was found that the incidence rate increased approximately as $(t - 19)^{4 \cdot 5}$ where $t$ is the age in weeks. Assuming the first hypothesis to be true and assuming a constant latent period $L$ of less than 40 weeks we would still have expected more tumours in the late-painted groups than in the young-painted groups (see Table III)

TABLE III.—*Expected Numbers of Infiltrating Carcinomas Assuming Various Latent Periods, Hypothesis* 1 *and an Incidence Rate Proportional to* $(t - 19)^{4\cdot5}$

| Latent period $L$ | Expected young | Expected old |
|---|---|---|
| 0–10 | 2·87 | 13·13 |
| 14 | 3·04 | 12·96 |
| 18 | 3·29 | 12·71 |
| 22 | 3·62 | 12·38 |
| 26 | 4·10 | 11·90 |
| 30 | 4·71 | 11·29 |
| 34 | 5·47 | 10·53 |
| 38 | 6·42 | 9·58 |

which is totally at variance with the observed numbers of 0 and 16 carcinomas respectively. This conclusion is not strongly dependent on the values 19 and 4·5; it is also obtained if other plausible values of $w$ and $k$ are used, e.g. 34 and 3·5. The only possible escape for hypothesis 1 now is to suggest that the latent period is not constant but increases with age.

The examination of this suggestion must await experiments involving stopping painting at various ages; if it is true, then there would be very little benefit to be obtained from stopping painting after the first year or so. This may be true, but it is at variance with the effects of cessation of smoking in humans where a rapid benefit is obtained (Doll and Hill, 1964). The data therefore suggest very strongly that the first hypothesis is to be rejected.

The group painted from the ninth week of life have, at age $t$ weeks, been exposed to the carcinogen for $(t - 9)$ weeks, whereas the old-painted group have, at age $t$ weeks, been exposed for only $(t - 68)$ weeks. If the incidence rate at age $t$ in the young-painted group is $b(t - 19)^{4\cdot5}$ then, if the incidence rate at age $t$ depends only on the cumulative past experience of the carcinogen, the incidence rate in the old-painted group should be $b(t - 78)^{4\cdot5}$ at age $t$. Using this, the expected numbers of carcinomas under the second hypothesis can be calculated: these are 0·5 in the old-painted group and 15·5 in the young-painted group, which conform excellently with the observed numbers 0 and 16 of carcinomas.

### REFERENCES

DAVIES, R. F. AND DAY, T. D.—(1969) *Br. J. Cancer*, **23**, 363.
DAY, T. D.—(1967) *Br. J. Cancer*, **21**, 56.
DOLL, R. AND HILL, A.—(1964) *Br. med. J.*, i, 1460.
PIKE, M. C.—(1966) *Biometrics*, **22**, 142.